# Implementation of the robotic abdominal phase during robot-assisted minimally invasive esophagectomy (RAMIE): results from a high-volume center

**DOI:** 10.1007/s00464-022-09681-1

**Published:** 2022-10-06

**Authors:** E. M. de Groot, L. Goense, B. F. Kingma, J. W. van den Berg, J. P. Ruurda, R. van Hillegersberg

**Affiliations:** Department of Surgery, University Medical Center Utrecht, Heidelberglaan 100, POBOX 85500, 3508 GA Utrecht, Netherlands

**Keywords:** RAMIE, Esophagectomy, Abdominal phase, Learning curve, Robot

## Abstract

**Background:**

Evidence on the added value of robotic-assistance in the abdominal phase during esophagectomy is scarce. In 2003, our center implemented the robotic thoracic phase for esophagectomy. In November 2018 the robot was also implemented in the abdominal phase. The aim of this study was to evaluate the implementation of the abdominal phase during robot-assisted minimally invasive esophagectomy (RAMIE).

**Methods:**

Consecutive patients who underwent full RAMIE with intrathoracic anastomosis for esophageal cancer were included. Patients were extracted from a prospectively maintained institutional database. A cumulative sum (CUSUM) analysis was performed for abdominal operation time and abdominal lymph node yield. Intraoperative, postoperative and oncological outcomes including collected lymph nodes per abdominal lymph node station were reported.

**Results:**

Between 2018 and 2021, 70 consecutive patients were included. The majority of the patients had an adenocarcinoma (*n* = 55, 77%) and underwent neoadjuvant chemo(radio)therapy (*n* = 65, 95%). The median operative time for the abdominal phase was 180 min (range 110–233). The CUSUM analysis for abdominal operation time showed a plateau at case 22. There were no intraoperative complications or conversions during the abdominal phase. The most common postoperative complications were pneumonia (*n* = 18, 26%) and anastomotic leakage (*n* = 14, 20%). Radical resection margins were achieved in 69 (99%) patients. The median total lymph node yield was 42 (range 23–83) and the median abdominal lymph node yield was 16 (range 2–43). The CUSUM analysis for abdominal lymph node yield showed a plateau at case 21. Most abdominal lymph nodes were collected from the left gastric artery (median 4, range 0–20).

**Conclusions:**

This study shows that a robotic abdominal phase was safely implemented for RAMIE without compromising intraoperative, postoperative and oncological outcomes. The learning curve is estimated to be 22 cases in a high-volume center with experienced upper GI robotic surgeons.

Robot-assisted minimally invasive esophagectomy (RAMIE) was introduced in 2003 and allows for precise dissection by offering three-dimensional vision, motion scaling and articulating instruments [1]. Recently, the ROBOT trial demonstrated superiority of RAMIE over open esophagectomy [2]. Research on the added value of a robotic system during esophagectomy has mainly focused on the thoracic phase while the added value in the abdominal phase has rarely been reported [3]. In our center, the da Vinci® robotic system (Intuitive Surgical Inc, Sunnyvale, CA) was initially used for the thoracoscopic phase only, as the first Standard-SI systems were not suitable for the abdominal phase due to the need for re-docking during the multiquadrant surgery that is required for dissection of the duodenum, greater gastric curvature, and hiatus. In addition, there were no robotic endowristed sealing instruments available. The dissection of the greater curvature along the gastroepiploic vessels with a rigid robotic ultrasonic scalpel did not add to conventional laparoscopic dissection. With the newest generation robot (Xi®) and the recently introduced robotic bipolar coagulator (vessel sealer®), these limitations have been solved and the robotic abdominal phase could provide technical benefits. However, little has been published about the potential benefits of robotic versus conventional minimally invasive surgery during the abdominal phase of RAMIE. In addition, evidence on the learning curve for the abdominal phase during RAMIE is scarce and only few studies have published on this topic [4, 5]. As surgeons are increasingly willing to adopt full RAMIE as their preferential technique, knowledge about the learning curve for the abdominal phase of RAMIE is important for proctoring programs in order to safely implement the robotic system in the abdominal phase. Therefore, the aim of this study was to evaluate the implementation of the abdominal phase during RAMIE in a high-volume center with experienced upper GI robotic surgeons.

## Materials and methods

### RAMIE experience

**Fig. 1:**
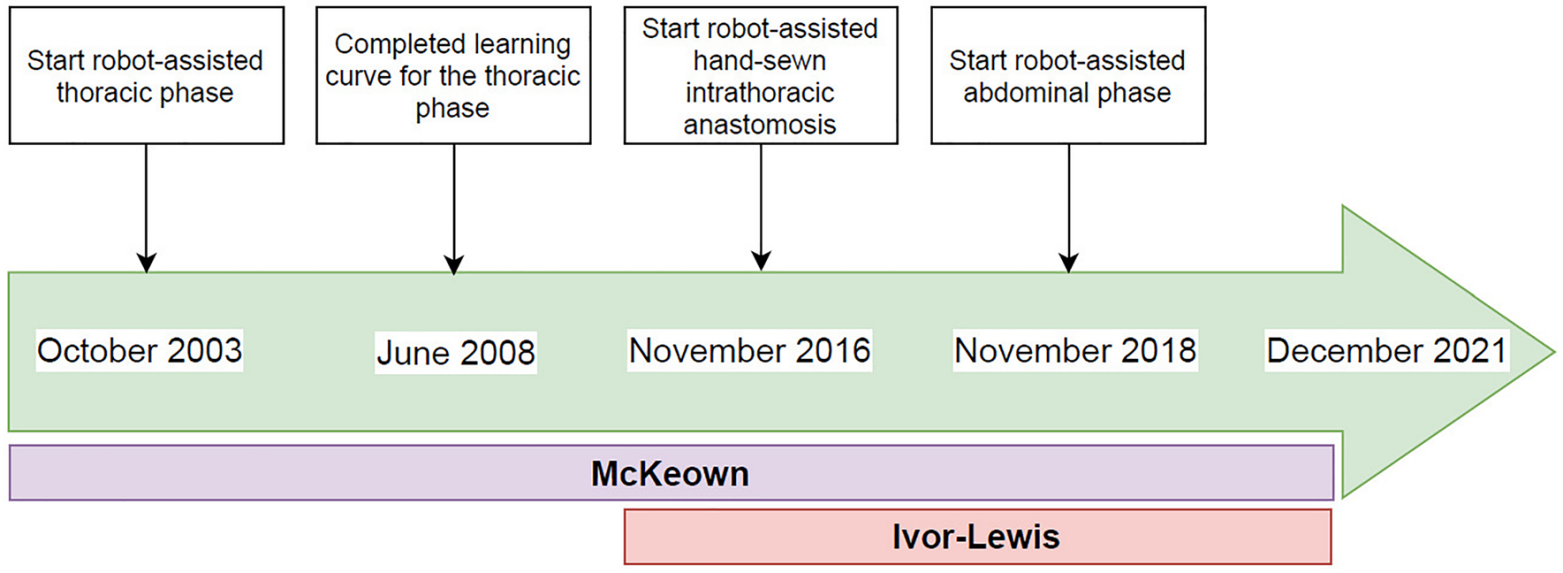
Timeline for the completed milestones for RAMIE

Robot-assisted minimally invasive esophagectomy (RAMIE) was implemented in 2003 in the University Medical Center Utrecht [6]. A timeline for key milestones in the development of RAMIE is shown in Fig. 1. Initially, only McKeown procedures were performed with a robot-assisted thoracic phase combined with conventional laparoscopy during the abdominal phase, except for the first 13 cases which involved laparotomies. The learning curve for the robot-assisted thoracic phase was completed in 2008 as published previously [7]. A robot-assisted hand-sewn anastomosis was introduced in 2016. From than onwards, in general patients with distal esophageal cancer underwent Ivor-Lewis esophagectomy and patients with mid or upper esophageal cancer underwent McKeown esophagectomy. In November 2018, the robot was also implemented during the abdominal phase. From then on, all consecutive patients underwent full RAMIE. All RAMIE procedures were carried out by JR and RvH. From October 2020 onwards, which corresponds with case 43, a new surgeon was proctored during the abdominal phase of RAMIE.

### Patient population

In this study, all consecutive patients who underwent full RAMIE with intrathoracic anastomosis for esophageal cancer between November 2018 and December 2021 were included. Patients were extracted from a prospectively maintained database. There were no exclusion criteria defined for this study. The institutional review board approved this study and the need for informed consent was waived.

### Surgical procedure

**Fig. 2:**
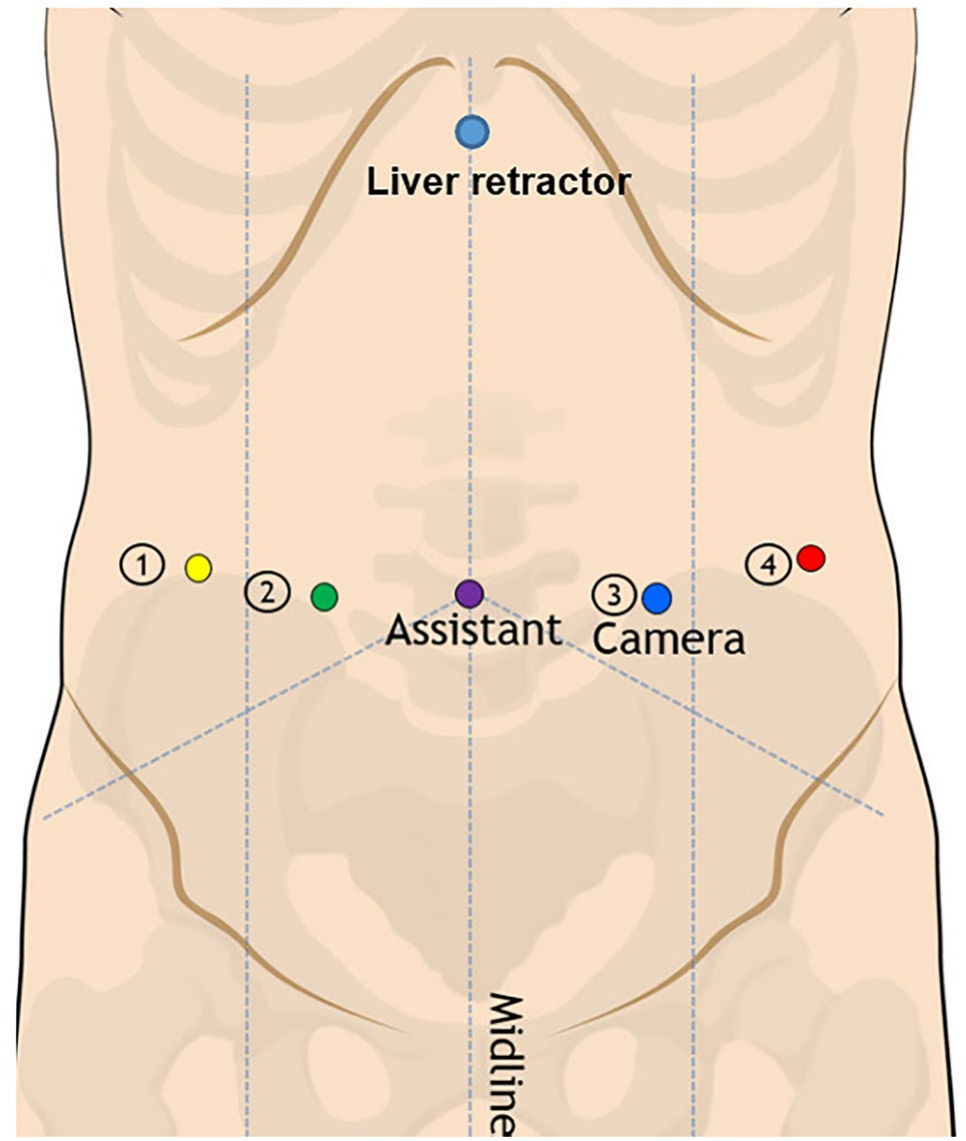
Abdominal port position for RAMIE; 4 robotic ports are inserted (3 × 8 mm and 1 × 12 mm), a 5 mm incision subxiphiodal for the liver retractor and a 10 mm incision for the assistant port

**Fig. 3:**
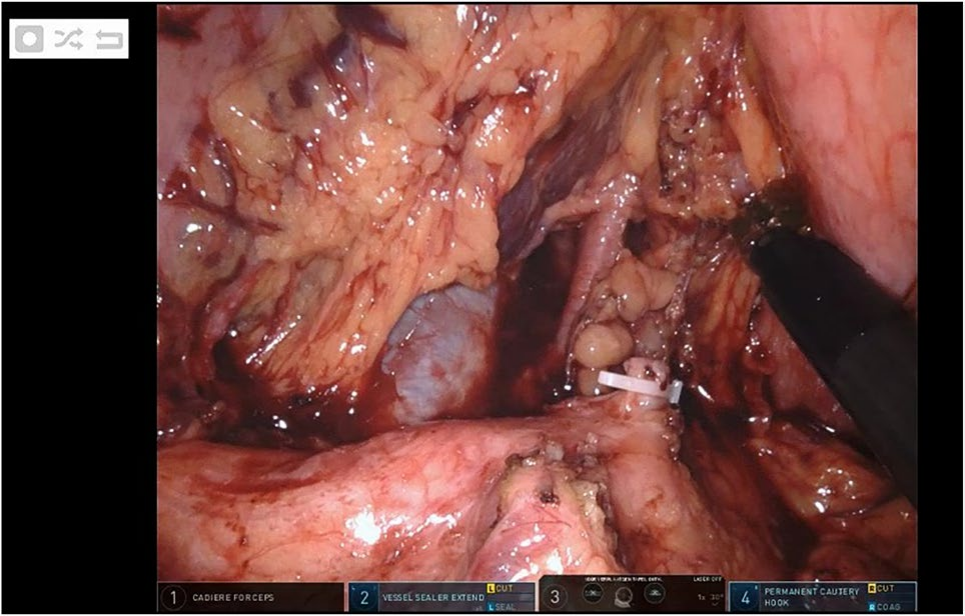
Robotic lymph node dissection of the celiac trunk (Station 9), the splenic artery (Station 11) and the hepatic artery (station 8) during RAMIE

RAMIE consisted of a two-field lymphadenectomy, gastric conduit reconstruction and intrathoracic hand-sewn anastomosis. The robot-assisted abdominal phase follows the same surgical steps as a laparoscopically performed phase [3]. The patient was placed in supine position after which 4 robotic ports were placed (3 × 8 mm and 1 × 12 mm) and a 5 mm incision subxiphiodal for the liver retractor and a 10 mm incision for the assistant port (Fig. 2). First, the lesser omentum was opened and dissection took place towards the crus with the Cautery Hook. Hereafter, the greater omentum was opened and the dissection was guided toward the short gastric vessels which were transected with the Vessel Sealer. The procedure continued with the dissection of the celiac trunk. The left gastric vein was transected with the vessel sealer and the left gastric artery with a hem-o-lock. The abdominal lymphadenectomy consisted of a dissection over the celiac trunk (Station 9), the splenic artery (Station 11) and the hepatic artery (station 8) (Fig. 3). The gastric conduit was created with an endowristed stapler device. From December 2018, a robotic stapler device was used. From January 2019, all patients routinely received a feeding jejunostomy which concluded the abdominal phase. In general, no fluorescence techniques were used in the abdominal phase. However, indocyanine green was routinely used in the thoracic phase to determine the location for the anastomosis at the gastric conduit [8]. From March 2019, all lymph nodes were collected separately in different containers according to the LOGICA study protocol [9]. The robot-assisted thoracic phase has been described in detail previously [10]. In summary, patients were placed in a semi-prone position after which 4 robotic arms were inserted and 1 assistant port. The esophagus was mobilized and a full mediastinal lymphadenectomy was performed. The gastric conduit was positioned in the esophageal bed. All patients had a hand-sewn robot-assisted intrathoracic anastomosis which was created with an end-to-side technique [11]. In general, all patients underwent cruroplasty with 2 independent sutures that concluded the thoracic phase.

### Outcomes

The primary outcome was the learning curve for the duration of the abdominal phase. The duration of the abdominal phase was defined by the time between the first incision and the moment that all incisions were closed. The docking time of the robotic system was not registered. Learning curve analyses were performed for the abdominal operating time (in minutes). Secondary outcomes were intraoperative complications, conversion rate, postoperative complications according to the definitions of the Esophagectomy Complications Consensus Group [12], length of hospital stay and in-hospital mortality. Oncological outcomes as lymph node yield and resection margins (R0 = margins not involved, R1 = one or more margins involved) were also extracted. Pneumonia was defined according to the Uniform Pneumonia Score [13]. All patient characteristics and perioperative outcomes were prospectively registered in a database. Complications were discussed and registered during a weekly multidisciplinary team meeting.

### Statistics

The learning curve for abdominal operation time and abdominal lymph node yield were demonstrated with cumulative sum (CUSUM) analyses (CUSUM = outcome measure of a single patient—mean outcome measure of the total cohort) [7, 14]. CUSUM allows for identifying the length of the learning curve per case instead of analyzing cohorts, resulting in a more precise analysis of the learning curve. The CUSUM formula provides a positive, neutral or negative outcome which is plotted against the mean of the total cohort. This is shown in a graph with the consecutive cases on the horizontal axis and the outcome of the CUSUM formula on the vertical axis. For abdominal operation time, the plateau phase was defined at the point when the operation time started to decrease and for the lymph node yield when the yield increased. Categorical variables were shown as counts with percentage. Continuous variables were shown as means with standard deviation or medians with range. Statistical analyses were performed by using SPSS 25.0 (IBM).

## Results

### Patients

Between November 2018 and December 2021, 70 consecutive patients underwent full RAMIE with intrathoracic anastomosis. Baseline characteristics are shown in Table 1. The majority of the patients had an adenocarcinoma (*n* = 55, 79%) and received neoadjuvant chemoradiotherapy (n = 63, 90%).

**Table 1:** Baseline values and treatment characteristics of the initial case series of 70 patients who underwent full RAMIE

Age, years (median, range)	66 (39–81)
Gender	
Male	53 (76)
Female	17 (24)
ASA score	
1	2 (3)
2	34 (49)
3	33 (47)
4	1 (1)
Tumor histology	
Adenocarcinoma	55 (77)
Squamous cell carcinoma	11 (17)
Other	4 (6)
Neoadjuvant therapy	
Chemoradiotherapy	63 (90)
Chemotherapy	2 (3)
None	5 (7)
Pathological T stage	
Tx	2 (3)
T0 (complete response)	19 (27)
T1	12 (18)
T2	9 (13)
T3	27 (39)
T4a	1 (1)
Pathological N stage	
N0	39 (56)
N1	20 (29)
N2	7 (10)
N3	4 (6)

### Intraoperative outcomes

**Fig. 4:**
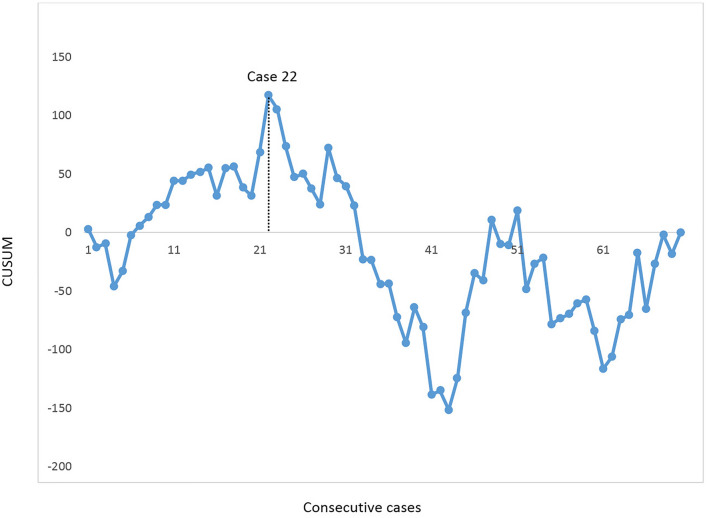
CUSUM learning curve analysis for *abdominal operating time* in the first 70 consecutive patients who underwent full RAMIE. The black dotted line demonstrates the plateau

The abdominal operation time was reported in all cases. The median abdominal operating time was 180 min (range 110–233). A CUSUM curve of the abdominal operation time is shown in Fig. 4. The plateau phase occurred at case 22, indicating that the abdominal operation time started to decrease. Total blood loss during RAMIE was median 250 ml (range 100–850). There were no intraoperative complications or conversions during the abdominal phase. During the thoracic phase, 1 complication and 1 conversion occurred. One patient had a bleeding from the subclavian vein which was resolved with a hemoclip. In 1 patient the right lung could not be desufflated requiring conversion to create adequate surgical exposure.

### Postoperative outcomes

Postoperative complications are demonstrated in Table 2. Out of 70 patients, 22 (31%) patients developed a postoperative complication Clavien Dindo grade 3 or higher. Anastomotic leakage was diagnosed in 14 patients (20%) and considered grade 1 in 3 patients (4%), grade 2 in 8 patients (11%) and grade 3 in 3 patients (4%). Chyle leak occurred in 5 patients (7%) and pneumonia was diagnosed in 18 patients (26%). The median hospital stay was 12 days (range 6–119). One patient (1%) died in the hospital of aspiration due to mechanic ileus.

**Table 2:** Intraoperative and postoperative outcomes of 70 patients who underwent full RAMIE with intrathoracic anastomosis

Duration abdominal phase, minutes (median, range)	180 (110–233)
Blood loss, milliliters (median range)	250 (100–850)
Intraoperative complications	
Abdominal phase	0
Thoracic phase	
Bleeding subclavian vein	1 (1)
Inability to collapse the right lung	1 (1)
Conversions, number (%)	
Thoracic phase	1 (1)
Average total lymph node yield	43 (23–87)
Resection margin	
R0	69 (99)
R1	1 (1)
Postoperative complications	
Any Clavien Dindo grade > 3	22 (31)
Pneumonia	18 (26)
Anastomotic leakage	14 (20)
Chyle leakage	5 (7)
Ileus	2 (3)
Hospital stay, days (median, range)	12 (6–119)
Mortality, number (%)	
In-hospital	1 (1)

### Oncological outcomes

**Fig. 5:**
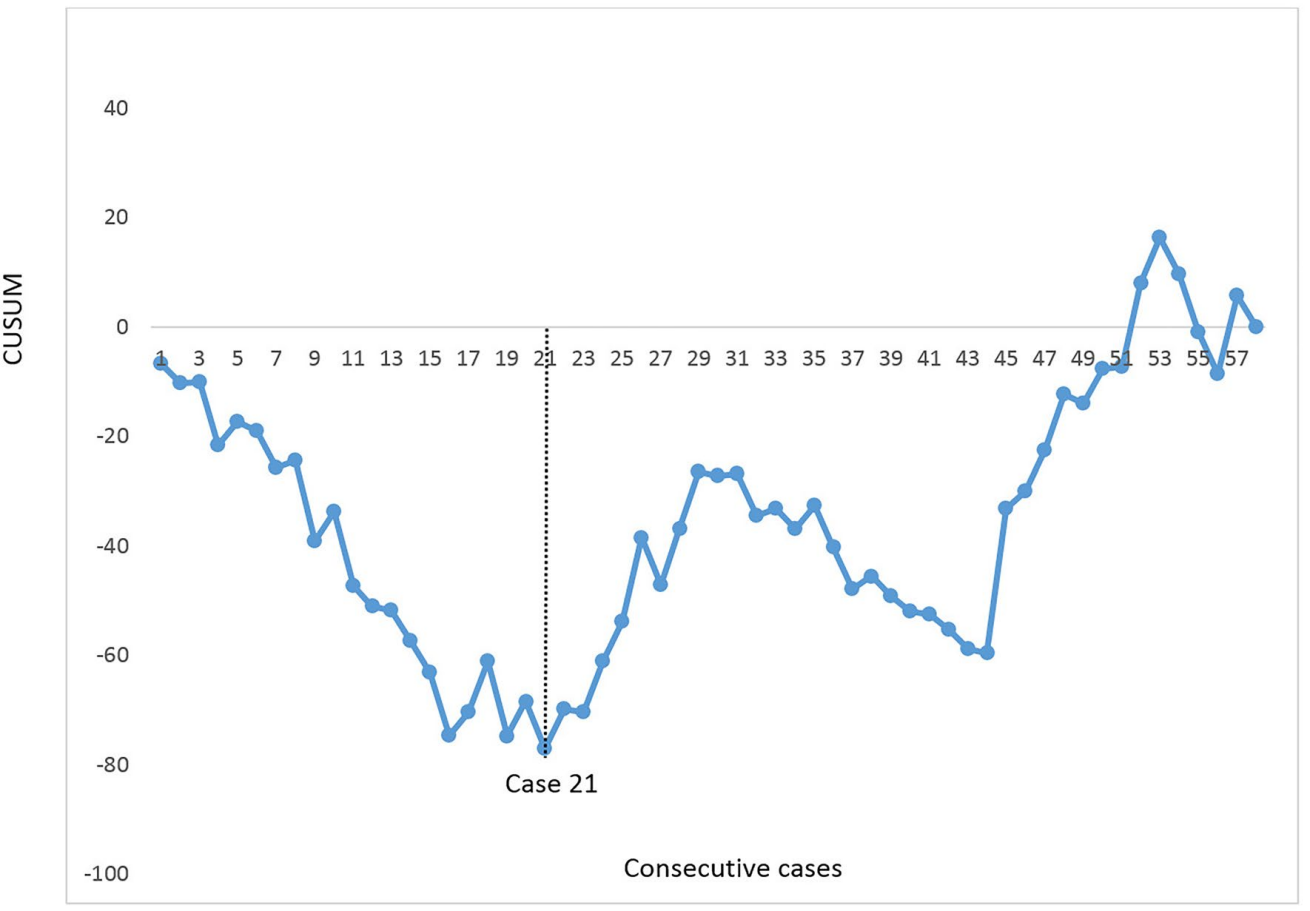
CUSUM learning curve analysis for *abdominal lymph node yield* in 58 consecutive patients who underwent full RAMIE. The black dotted line demonstrates the plateau

A complete resection (i.e., R0) was achieved in 69 patients (99%). The median total lymph node yield was 42 lymph nodes (range 23–83). From March 2019, lymph nodes were collected separately per station according to the LOGICA study protocol. Hence, in 58 patients the lymph node stations were collected separately which is demonstrated in Table 3. From that group, the median abdominal lymph node yield was 16 (range 2–43) and the median thoracic lymph node yield 20 (range 2–46). A median of 6 lymph nodes (0–22) were left attached to the resection specimen or were other lymph node stations. The majority of the abdominal lymph nodes were collected near the left gastric artery (median 4, range 0–20), followed by the hepatic artery (median 3, range 0–9), paracardial (median 2, range 0–12), celiac trunk (median 1, range 0–8) and the splenic artery (median 1, range 0–9). A CUSUM was generated for the abdominal lymph node yield and shown in Fig. 5. After case 21, a plateau phase was observed after which the lymph node yield started to increase.

**Table 3:** Dissected abdominal lymph nodes per station in 58 patients who underwent full RAMIE with intrathoracic anastomosis

Total lymph node yield, median (range)	
Abdominal Thoracic	16 (2–43) 21 (0–46)
Other/resection specimen	7 (0–22)
Abdominal lymph node yield per station, median (range)	
Left gastric artery	4 (0–20)
Hepatic artery	3 (0–9)
Celiac trunk	1 (0–8)
Splenic artery	1 (0–9)
Paracardial	2 (0–12)

## Discussion

This study reports the implementation of a robot-assisted abdominal phase in 70 consecutive patients who underwent RAMIE for cancer in a high-volume center. There were no intraoperative complications or conversions during the abdominal phase and satisfying postoperative and oncological outcomes were achieved, implying that the implementation of the robot-assisted abdominal phase went safe.

The median abdominal operation time was 180 min (range 110–233). A plateau for abdominal operation time occurred after 22 cases, demonstrated by the CUSUSM analysis. Previous analysis of the learning curve of the thoracic phase by our study group demonstrated that 70 cases were needed to reach the first plateau of proficiency and 24 cases when a structured training program was followed [7]. The pre-existing experience of the surgical team in robot-assisted esophagectomy likely resulted in a shorter learning curve compared to the thoracic phase [15]. In addition, the more than 10 year experience in laparoscopy was probably an advantage compared to a transition from laparotomy to a robot-assisted abdominal phase. Nevertheless, after 43 cases, an increase in operation time was observed. This may be caused by proctoring, as parts of the abdominal phase were performed by fellows in the context of proctoring. Only few other studies demonstrated CUSUM analysis for abdominal operating time during RAMIE. A recent study demonstrated a similar CUSUM for abdominal operation time and showed that the plateau was reached after case 24 [5]. Another study reported a plateau after case 14 for the console time of the robot-assisted abdominal phase.

A recent publication from the Upper GI International Robotic Association (UGIRA) study group demonstrated that only half of the RAMIE procedures are performed fully robotic while the other half consists of a robot-assisted thoracic phase combined with laparoscopy or laparotomy [16]. The added value of a robotic system over laparoscopy during RAMIE is still under debate, as no studies exist specifically comparing conventional laparoscopy to a robot-assisted abdominal phase in RAMIE. However, there are several potential benefits of a robot-assisted abdominal phase. First, the abdominal lymph node dissection around delicate structures (e.g. celiac trunc, hepatic artery, splenic artery) is facilitated by the use of a robotic system. Technical advantages of the robotic system such as a stable camera view, tremor reduction and a fourth arm facilitate a safe though thorough lymph node dissection in this area. In this context several propensity-score matched studies and one randomized control trial have compared abdominal lymph node yield after full RAMIE to full conventional minimally invasive esophagectomy (MIE). All 5 propensity-score matched studies showed a higher abdominal lymph node yield in the robot group, which was statistically significant in 2 studies [17–21]. Results of these studies are summarized in Table 4. The randomized controlled trial comparing 181 full RAMIE patients to 177 full MIE patients with squamous cell carcinoma did not show a difference in abdominal lymph node yield [22]. It must be noted that a median of 7 abdominal lymph nodes (range 4–10) were harvested in both groups which is relatively low especially in contrast with the current study with a median abdominal lymph node yield of 16 (range 2–43). That study might not have focused on the abdominal lymph node making it hard to draw conclusions on the added value of the robot for that part of the procedure.

**Table 4:** Study characteristics and abdominal lymph node yield studies comparing full RAMIE to full MIE

Author	Study design	Procedure	Group 1	Group 2	Patiens *n* =	Abdominal LNY		
						Robotic	Laparoscopic	*p* value
Deng et al. [14]	PSM	McKeown	Fully robotic	Thoracoscopy-laparoscopy	42 vs. 42	10.8 ± 8.1	7.7 ± 4.8	**0.041**
Deng et al. [15]	PSM	McKeown	Fully robotic	Thoracoscopy-laparoscopy	52 vs. 52	9.7 ± 6.4	7.3 ± 5.1	**0.042**
Zhang et al. [16]	PSM	Ivor Lewis	Fully robotic	Thoracoscopy-laparoscopy	66 vs. 66	8.9 ± 6.7	7.3 ± 5.9	0.198
Yang et al. [17]	PSM	McKeown	Fully robotic	Thoracoscopy-laparoscopy	271 vs. 271	7.9 ± 4.8	6.8 ± 3.6	0.237
Xu et al. [18]	PSM	McKeown	Fully robotic	Thoracoscopy-laparoscopy	292 vs. 292	9.23 ± 3.57	9.02 ± 1.85	0.373
Yang et al. [19]	RCT	McKeown	Fully robotic	Thoracoscopy-laparoscopy	181 vs. 177	7 (4–10)*	7 (4–10)*	0.274

All outcomes are reported as mean with standard deviation except for *, which is a median with range

All number of patients from PSM studies are the numbers in the matched cohorts

*NR* not reported, *LNY* lymph node yield, *PSM* propensity-score matched, *RCT* randomized controlled trial

Although only few studies focused on the abdominal lymph node yield during RAMIE, multiple studies focused on lymph node yield during robot-assisted gastrectomy which has a similar lymphadenectomy. Those studies stated that a robot-assisted lymph node dissection might be superior over laparoscopy which is promising for RAMIE as well [23–25]. However, comparing lymph node yield between studies is challenging, since this outcome not only depends on the dissection but also on the methods used for pathology assessment of the resection specimen. Lymph nodes that are separately presented per station instead of en-bloc resections are known to improve lymph node yield, which was the case in the current study [26].

A second potential benefit of a robot-assisted abdominal phase over laparoscopy is that it might be cost-reducing, especially if the thoracic phase is already performed with robotic-assistance. In that case, the same instruments used during the thoracic phase can be used for the abdominal phase instead of a new set of 4–5 laparoscopic instruments, leading to a cost reduction. In addition, recent studies showed that the abdominal operation time for full RAMIE is shorter compared to hybrid RAMIE [22, 27].

Several different analyses are reported to demonstrate the learning curve of RAMIE including CUSUM analyses [29]. A benefit of CUSUM analyses is that it allows for an outcome per individual case, and not per group. Patient outcomes as well as procedure-related outcomes including operation time and blood loss are used for CUSUM analysis [23]. Since patient outcomes generally have a multifactorial etiology, procedure-related outcomes might be more suitable to determine the learning curve. Therefore, this study performed CUSUM analysis for abdominal operation time and lymph node yield.

A strength of this study is the unique level of detail of the dissected abdominal lymph node stations, collected in separate packages in the majority of the patients. In addition, the data was collected in a prospectively maintained database. A limitation might be that the procedures were performed by 2 surgeons who both already completed the learning curve for the robot-assisted thoracic phase. Therefore, the results might not be generalizable to surgeons without robotic experience. On the other hand, it allows for purely investigating the learning curve of the robot-assisted abdominal phase without involving other learning curves.

The results in the current study showed that a robotic system was safely implemented in the abdominal phase during RAMIE achieving satisfying outcomes. In addition, the learning curve for the robot-assisted abdominal phase was relatively short, likely due to the experience in laparoscopy and because the learning curve for robot-assisted thoracic phase was already completed. Future studies should investigate whether a robotic system is of added value for the abdominal phase over laparoscopy during RAMIE. In order to truly compare a robotic abdominal phase to laparoscopy, 2 cohorts without a learning curve should be compared.
